# Challenges of implementing Mark-recapture studies on poorly marked gregarious delphinids

**DOI:** 10.1371/journal.pone.0198167

**Published:** 2018-07-11

**Authors:** Krista Hupman, Karen A. Stockin, Kenneth Pollock, Matthew D. M. Pawley, Sarah L. Dwyer, Catherine Lea, Gabriela Tezanos-Pinto

**Affiliations:** 1 National Institute of Water and Atmospheric Research, Wellington, New Zealand; 2 Coastal-Marine Research Group, Institute of Natural and Mathematical Sciences, Massey University, Auckland, New Zealand; 3 Department of Applied Ecology, North Carolina State University, Raleigh, North Carolina, United States of America; Liverpool John Moores University, UNITED KINGDOM

## Abstract

Population parameters of poorly marked gregarious species are difficult to estimate. This is the case for common dolphins (*Delphinus* sp.), a genus known for its lack of distinctive marks resulting in a low mark ratio. Furthermore, the widespread nature of common dolphins results in low recaptures. We developed reliable photo-identification protocols to ensure accurate identification of individuals in the Hauraki Gulf, New Zealand. These protocols combined the use of nicks and notches and pigmentation patterns for identification and included the development of a distinctiveness threshold. The data were further stratified by the level of distinctiveness of each individual (as distinctive or highly-distinctive). Photo-identification surveys were conducted from January 2010 to December 2013. Mark-recapture techniques were implemented through a POPAN super-population approach to estimate seasonal apparent survival, capture probability and abundance of dolphins. A total of 2,083 unique adult common dolphins were identified, 51.3% were classified as D1 (highly distinctive; *n* = 1,069) and 48.7% as D2 (distinctive; *n* = 1,014). Of all individuals identified, 34.3% (*n* = 704) were re-sighted over subsequent years. The proportion of marked dolphins (when compared to unmarked dolphins) was 26.3% for D1 and 46.4% for D1 & D2, respectively. Apparent survival was estimated at 0.767 (CI = 0.694–0.827) for D1 animals, and 0.796 (CI = 0.729–0.850) for D1 & D2 combined. For D1 only, seasonal abundance varied from 732 (CI = 460–1,177) in autumn 2010 to 5,304 (CI = 4,745–5,930) in spring 2013. While the inclusion of D2 individuals may offer a more precise estimate of total abundance, the inability to determine additional sources of bias (for example, arising from under or overestimated mark ratios) meant that estimates for D1 individuals were deemed the least biased for this population. The photo-identification protocol, stratification of the data and steps taken to eliminate potential model violations provided a useful and novel approach to estimate population parameters for common dolphins. These approaches could be implemented for other large gregarious populations (≥500 individuals) of animals with poor natural markings.

## Introduction

Photo-identification (photo-id) has been widely implemented to estimate population parameters for coastal dolphins [1–20], however, it is less commonly applied to gregarious oceanic species [21–23]. This is, in part, because it is often impossible to photograph every individual for species that aggregate in large groups that are often widely dispersed. This may result in low capture probabilities that may lead to imprecise estimates [24]. Moreover, conducting offshore surveys is often challenging and expensive [25] given the resources required.

The use of opportunistic sampling has been increasingly recognised as providing an alternative means for scientific data collection on cetaceans [26–35]. Opportunistic sampling is relatively inexpensive and permits animals to be sampled at non-specific time intervals. This is a useful approach to sample species with heterogeneous distributions. However, opportunistic sampling does have limitations, that may include restricted search areas, limited time with focal groups, and inability to identify species or estimate group size. These limitations may violate capture-recapture assumptions, affect the type of analyses that can be conducted, or produce biased estimates that reflect the survey design instead of true ecological patterns [36]. It is for this reason that some photo-id studies combine opportunistic and dedicated sampling methods, to maximise data collection on cetaceans [37–40].

Several methods have been reported to uniquely identify free-ranging cetaceans allowing for individual recognition [41]. For example, a number of small cetacean species have been identified using temporary markings such as scars [42], although their unstable nature means they are less reliable over prolonged temporal scales [43]. More permanent methods of marking such as attachment [44] or genetic tagging [45] have also been used and are more reliable over prolonged periods. However, these methods tend to be more labour-intensive and expensive [43, 46]. The most frequently used technique to identify individual cetaceans is photo-id, whereby the recognition of individuals is based on their unique natural features or markings [41, 47–48]. This method is preferred because it is less invasive and provides a relatively inexpensive means to catalogue marked individuals within a population [46]. However, application of this method depends on animals possessing permanent natural features that allow for the unique identification of individuals.

In many delphinid photo-id studies, the most frequently used identifying features include nicks and notches present on the leading and trailing edges of the dorsal fin [41]. However, not all marked animals have an equal probability of being identified. Unequal identification can result from: difficulties in detecting particular features because of different levels of photographic quality (PQ) [49], variability in the level of individual distinctiveness as some animals have more distinctive identifying features than others (i.e. nicks and notches/pigmentation patterns) [50], and/or variations in an individual’s behaviour that may affect detectability (e.g. some animals are less prone to approach boats) [51]. For example, low quality photographs reduce the ability of identification as some features may not be visible [50, 52]. A number of studies have therefore, applied strict criteria to assess PQ and nick/notch distinctiveness (ND) to ensure unique individual identification is reliable [49, 53–54]. This is in an effort to reduce misidentification (false-negative errors bias abundance estimates high; false-positive errors bias abundance estimates low) or heterogeneity (biases abundance estimates low) [50].

The efficiency of photo-id also depends on the proportion of individuals within a population that exhibit sufficient marks allowing unique recognition—not all individuals observed within a population may have unique marks. Therefore, when estimating abundance, estimates need to account for the unmarked proportion of the population (i.e. those animals recorded with insufficient marks for individual identification). The mark ratio represents the proportion of individuals within a population that are marked relative to the total observed population [55–56]. Estimation of abundance can be challenging as both the mark ratio and the abundance of marked individuals need to be estimated. When such estimates are combined, two standard errors also become combined to estimate the SE of the total population (i.e. marked and unmarked), that therefore reduces precision. A low mark ratio occurs when there is a high proportion of unmarked individuals, that makes estimation of abundance more challenging because the standard error of the total abundance estimate will increase [57–58]. Of the many studies that have used photo-id to examine small delphinids [1–7, 10–20, 22–23, 38, 57, 59–88], mark ratios have been shown to vary greatly for different species and for different populations within the same species [5, 38, 59, 67, 72, 74, 87].

Short-beaked common dolphins (*Delphinus delphis*) are a pelagic species that can be difficult to identify as they are found in oceanic environments and are poorly marked [86]. Considering this, most published abundance estimates worldwide for *Delphinus* originate from aerial [89] or shipboard [90–91] surveys that count rather than identify (and recapture) individuals. The few published studies have applied photo-id [78–81, 86, 92], have mostly focused on calculating minimum estimates rather than total abundance and survival [81–82]. Furthermore, the process of cataloguing and matching large numbers of individuals in a large population can be time-consuming [82]. Mark-recapture (MRC) studies, do, however, have the advantage of yielding estimates of population parameters. The aim of this study was to present the challenges of implementing MRC methods to estimate population parameters for delphinids with a low mark ratio, in this case, common dolphins in the Hauraki Gulf (HG), New Zealand. Specific objectives were to: apply several reliable photo-id protocols to increase the identification rate; examine photo-id data using two different grades of ND to identify the most accurate estimates of population parameters, and; estimate apparent survival, capture probability, probability of entry, and abundance.

## Materials and methods

### Ethics statement

The New Zealand Department of Conservation is the government agency responsible for the protection and management of New Zealands wildlife and the designation of special areas of conservation. No specific permission or permit was required for the fieldwork/data collection, as the Hauraki Gulf (36° 10’ to 37° 10’ S, 174° 40 to 175° 30’ E) is a public area. The study did not involve the handling or management of dolphins, but instead involved photo-id. As this method is considered to be non-invasive, no permissions or permits were required for data collection for the common dolphin.

All research effort was collected in strict accordance with the New Zealand Department of Conservation’s recommendations for operating vessels around marine mammals, the Marine Mammals Protection Act 1978 and Marine Mammals Protection Regulations 1992.

### Field methods

**Fig 1:**
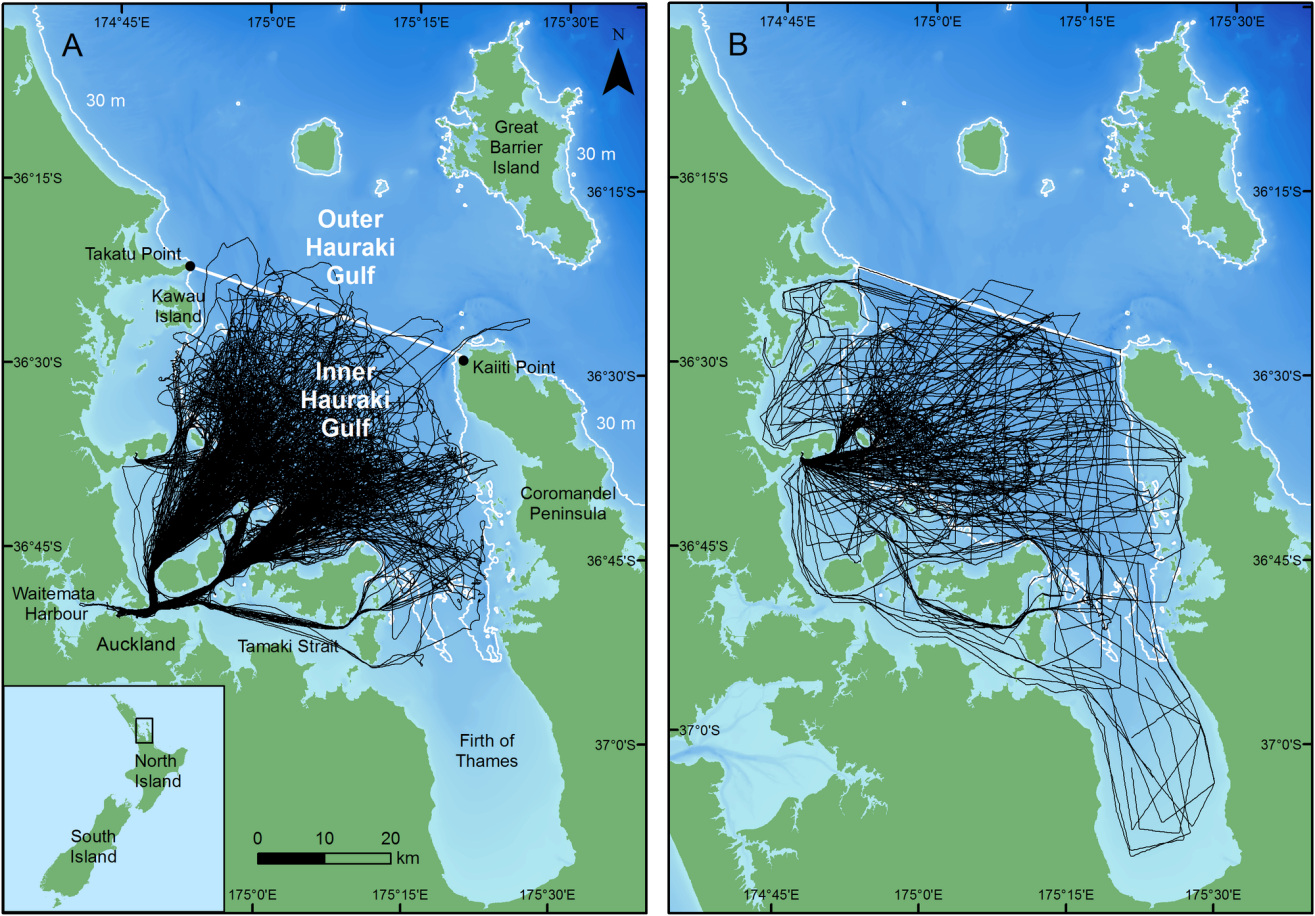
Survey tracks (black lines) of a) tour and b) research vessels in the inner Hauraki Gulf, New Zealand. The solid black line indicates the boundary between the inner and outer HG. The white and yellow lines indicate the 30 and 100 m isobaths, respectively. Bathymetry is depicted with darker shades of blue representing deeper waters (reprinted from NIWA under a CC BY license, with permission from NIWA original copyright 2012; [93]). Inset: Location of the Hauraki Gulf and other places referred to in the text in relation to the North Island of New Zealand.

Photo-id of common dolphins was collected in the HG, between January 2010 and December 2013. The HG is situated on the north-eastern coastline of the North Island, New Zealand (Fig 1). An arbitrary line between Takatu Point and Kaiiti Point was used to delineate between the inner and outer HG [31]. Surveys were only conducted in the inner HG (hereafter referred to as the study area/HG).

Observations of common dolphins were conducted during non-systematic surveys from three vessels: *Te Epiwhania* and *Aihe II*, both 5.5 m research vessels; and *Dolphin Explorer*, a 20 m tourism platform (Table 1). The average survey duration from all vessels was approximately eight hours (SE = 0.31). Surveys conducted were either opportunistic (where photo-id was not the only focus of surveys) or dedicated (where photo-id was only focus of surveys) in nature (Table 1). The number of observers remained constant regardless of the vessel. Non-systematic surveys were implemented to (i) reduce costs and (ii) maximise data collection given the large size of the study area (3,480 km^2^) and the different types/nature of vessels used for surveys.

**Table 1:** Description of vessels used for non-systematic surveys. The nature of the survey refers to the opportunistic (where photo-id was not the only focus of surveys) or dedicated (where photo-id was only focus of surveys).

Vessel	Type of vessel	Nature of survey	# Observers	Survey dates
*Te Epiwhania*	Research	Opportunistic	Two	Aut 2010—Win 2011
*Dolphin Explorer*	Tourism platform	Dedicated	Two	Spr 2011—Sum 2013/14
*Aihe II*	Research	Dedicated	Two	Sum 2012—Sum 2013/14

Common dolphins are distributed throughout the inner HG, though concentrated in the central inner HG region [94–95]. Consequently, all vessels allocated their search effort primarily within the central inner HG (Fig 1). Additionally, research effort focused in waters >30 m depth given that common dolphins are less frequently found in shallow inshore bays of the inner HG (such as the Waitemata Harbour, Tamaki Straight, and Firth of Thames; [94–95]).

All vessels departed from either the Viaduct Marina or Gulf Harbor Marina. The skipper of each vessel would subsequently assess the visibility and sea state within the central inner HG and assess where common dolphins were recently sighted (as noted on GPS plots). A route would then be planned based on areas of favorable weather conditions and recent sightings. Sightings of dolphins were shared amongst vessels (when operating at the same time), and were opportunistically provided via marine radio from other vessels within the HG. As there is only one tourism vessel operating in the HG, there was no network of other tourism operators available for retrieval of additional sighting information. In the case where dolphins had not been sighted within a day, the skipper would survey previous high-encounter areas. When weather permitted, an effort was made by the skipper of each vessel to cover areas that were not previously surveyed. The primary objective of each survey was to locate as many independent groups of common dolphins as possible to conduct photo-id.

Dolphins were classified as either immatures (neonates, calves, and juveniles) or adults [29] (S1 Fig). Only adults were included in the MRC analysis. This is because immature dolphins tend to be unmarked and stay with their mother until weaning [96], that may bias population estimates (i.e. dependant fates;51]. Group size was estimated visually by counting the number of individuals both surfacing and underwater (where water visibility allowed) and the best estimate [97–98] was used to determine group size categories and for mark ratio calculations. The best estimate represented a point estimate determined by the observer, according to the conditions at the time, taking into account the possibility of double counts [99].

### Photo-identification

One or two trained researchers conducted concurrent photo-id sessions from all vessels following standardised methods [41], using Nikon D90 and D7000 SLR cameras fitted with Nikon 100–300 mm and 100–400 mm zoom lenses, respectively. The number of researchers conducting photo-id sessions was dependant on dolphin group size and space availability on the tourism vessel. Dolphins located within a 100 m radius, moving in the same direction and (usually) engaged in the same activity were considered to be part of the same group [29]. Each group of dolphins photo-identified were referred to as an ‘encounter’. Photographs were taken when dolphins surfaced within 25 m of either vessel [23].Considering subtle nicks and notches may not be easily recognizable from both right and left sides of the dorsal fin, and given pigmentation remains inconsistent between sides, only the left side of the dorsal fin was photographed. Photo-id was randomly collected without biasing towards marked or unmarked individuals [41]. Regardless of group size, an attempt was made to photograph as many individuals within the group as possible. Dolphin groups were classified as either: *all captured* (AC), or *not all captured* (NAC). Groups were only considered AC when all animals in the group were photographed and the group size was ≤15 individuals. All other groups were classified as NAC. Photo-id was continued until all individuals within a group were photographed, except when dolphins showed avoidance behaviour (i.e. displacement from the vessel), conditions deteriorated (e.g. Beaufort Sea State ≤4, diminished light levels), or the tour platform terminated the encounter.

#### Grading and sorting of photo-identification images

Individual identification was based on natural dorsal fin markings, including nicks and notches on the leading and trailing edge of the left side of the dorsal fin (referred to as ‘marked individuals’) [41, 47]. Dorsal fin pigmentation patterns on the left side of the dorsal fin were used as a secondary identification feature since patterning was found to be stable for at least 11 years [98]. Photographs were graded (see below) and compared manually [23].

Each image was first assessed to determine the proportion of the dorsal fin in the frame. When the dorsal fin occupied <10% of the frame, it was automatically excluded from the analysis. Secondly, all images were graded according to PQ [4, 49], with the aim of minimising bias and reducing misidentifications. Each image was assigned a value based on the following categories: clarity and focus (scored as poor—1, reasonable—4, or excellent—9); degree of contrast (scored as 1, 3, or 9); orientation (angle; scored as poor—1, reasonable—2, or excellent—9), and; dorsal fin edge visibility (scored as poor/reasonable—1 or excellent—8) (S1 Table; adapted from [4] and. [49]). Values for each category were then summed to produce an overall image quality score, from poor to excellent (S2 Fig). Scores for each category were weighted so that inadequate quality in one category alone would ensure an image was rated as poor [4].

The depth of nicks and notches on the dorsal fin were measured both vertically and horizontally using ImageJ (version 1.48) [100]. The relative depth of the largest nick/notch was estimated by dividing the measured depth by the total length between the anterior and posterior insertions of the dorsal fin [101]. When the relative proportion was <10 or ≥10%, nicks and notches were classified as minor (<1) or major (≥1 cm), respectively (S3 Fig).

**Fig 2:**
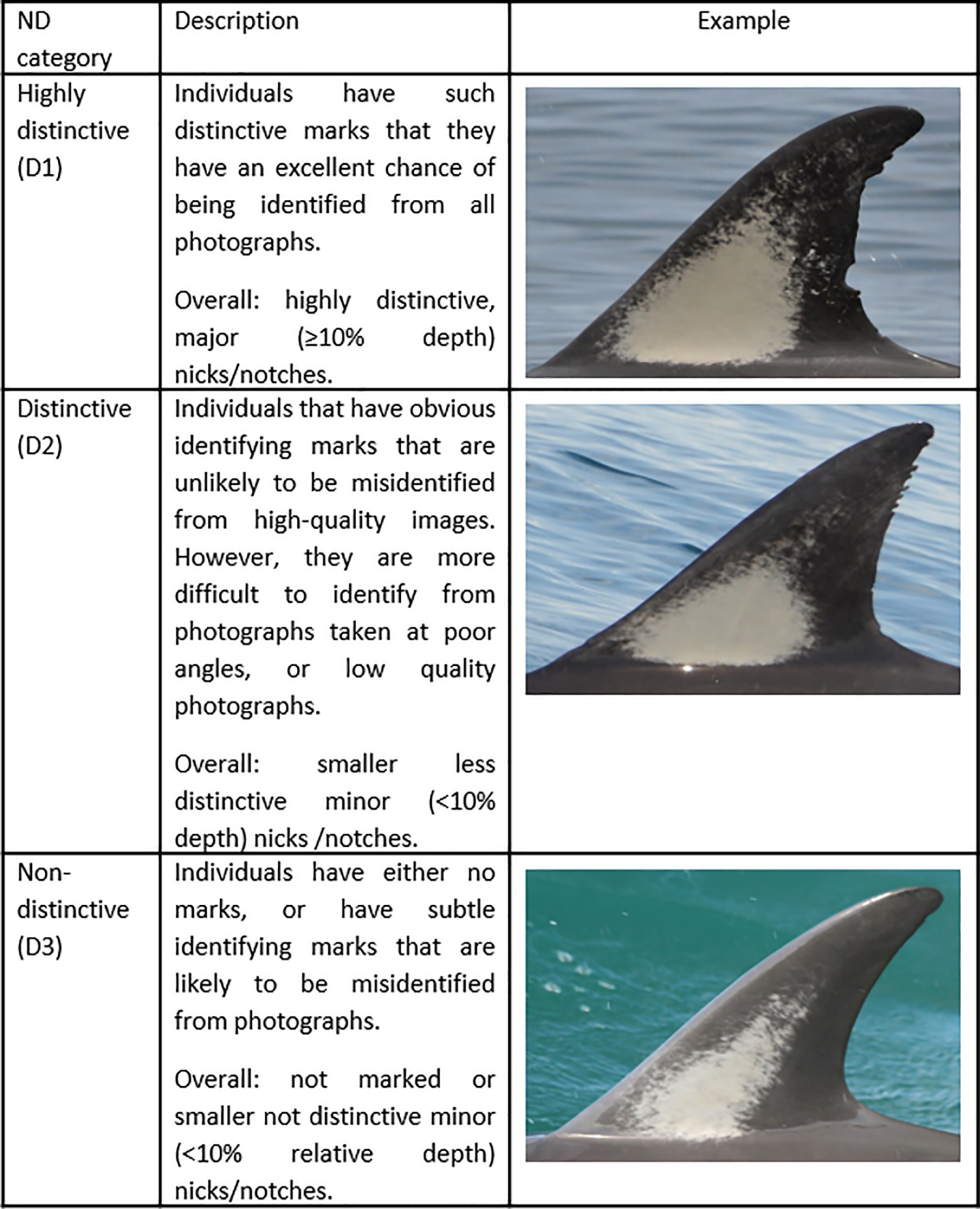
Nick/notch distinctiveness (ND) categories used to examine adult common dolphin (*Delphinus* sp.) images in the Hauraki Gulf, New Zealand. Individuals were classified as: highly distinctive (D1); distinctive (D2), or; non-distinctive (D3) [1, 4]. The relative depth of the largest nick/notch was determined by dividing the depth of the nick/notch (as measured on a photograph) by the total length of the base of the dorsal fin [101].

Following the assessment of nick/notch depth, each image was graded according to ND. Only the largest nick/notch on either the leading or trailing edge of each dorsal fin was used to classify ND under the following categories: a) highly distinctive (D1); b) distinctive (D2), and; c) non-distinctive (D3) (Fig 2) [1, 4, 23].

To use photo-id for poorly marked species, it is important to acknowledge that PQ and ND are not independent [53–54]. This is because D1 animals can be identified in images of lower PQ and vice versa [54]. The use of D1 individuals from lower PQ may either introduce or increase heterogeneity in capture probabilities. Therefore, it is recommended that the degree of distinctiveness that will be used is determined first, and then a decision is made on the image quality threshold necessary to recognise animals based on such a level of distinctiveness [54]. When examining ND in the present study, D1 individuals could only be used when PQ was rated as fair, good, or excellent quality (poor quality photographs were excluded). For D2 individuals, photographs were only used when they were rated as good or excellent quality (poor and fair quality photographs were excluded). When image quality criteria were met, images were referred to as ‘high quality’.

**Fig 3:**
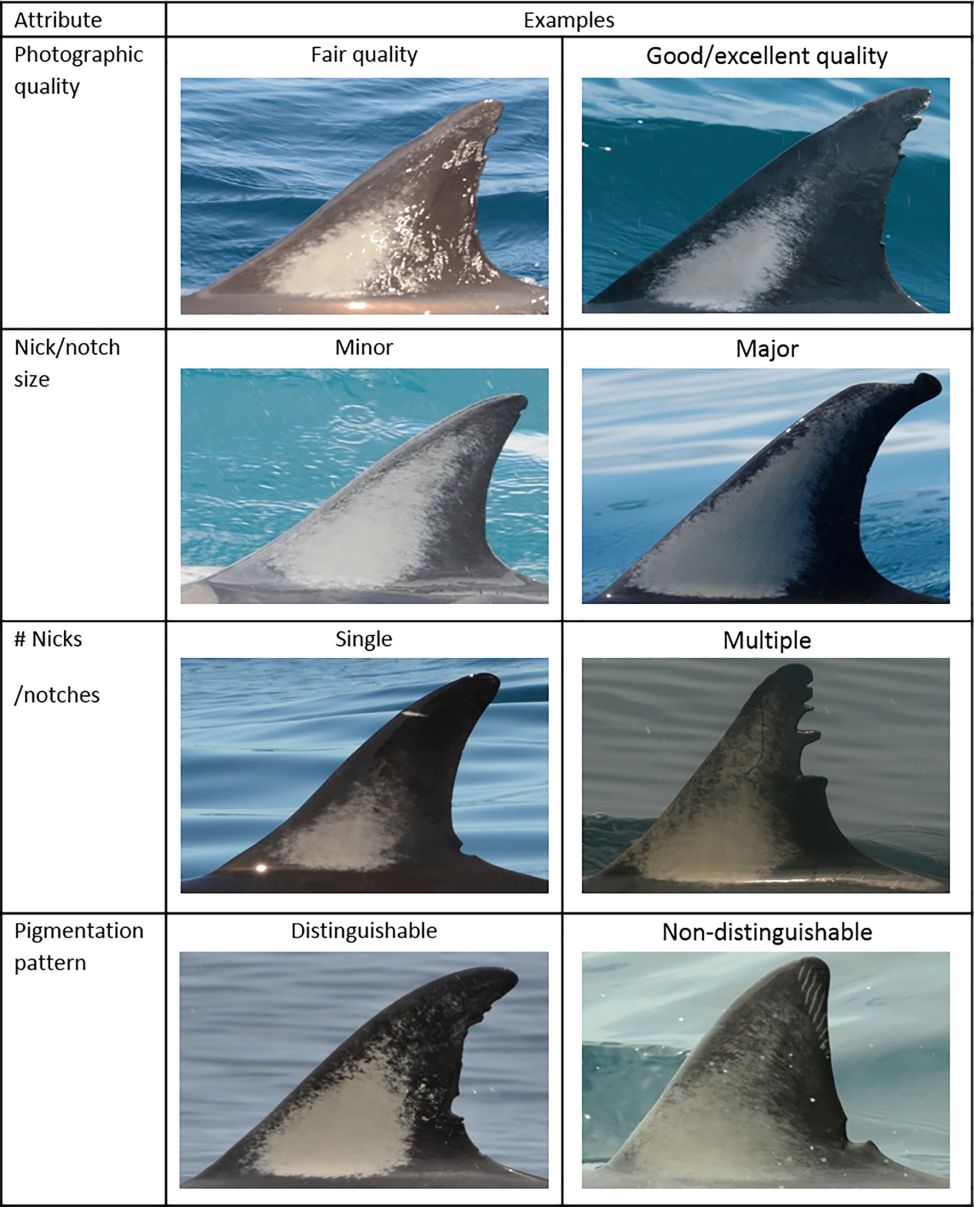
Attributes used to determine if an individual adult common dolphin (*Delphinus* sp.) was a distinctively marked individual (DMI).

**Fig 4:**
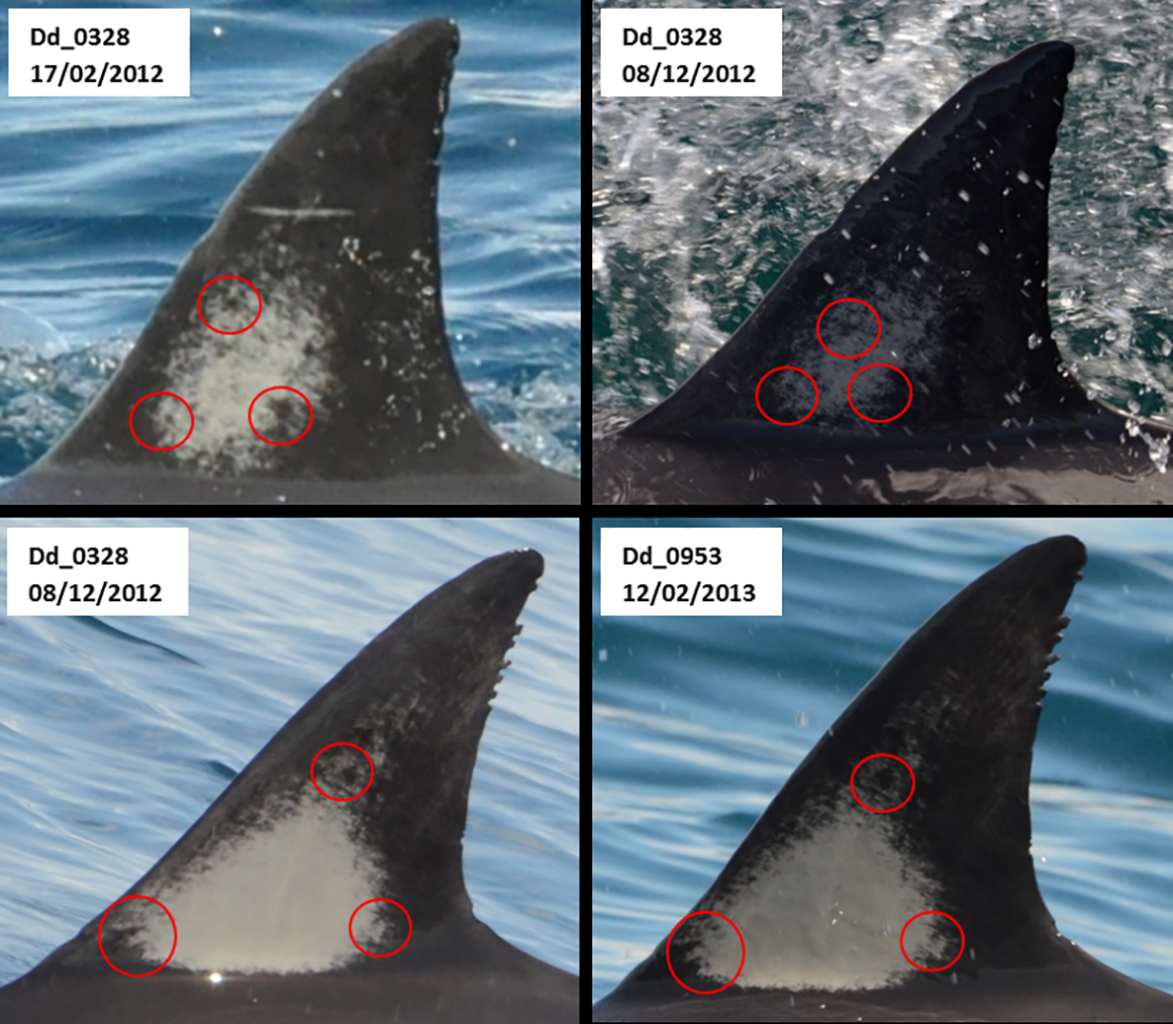
Examples of distinguishable pigmentation patterns for two adult common dolphins (*Delphinus* sp.) (Dd_0328 and Dd_0953) photographed in the Hauraki Gulf, New Zealand. Red circles highlight sections of distinctive corresponding pigmentation.

**Fig 5:**
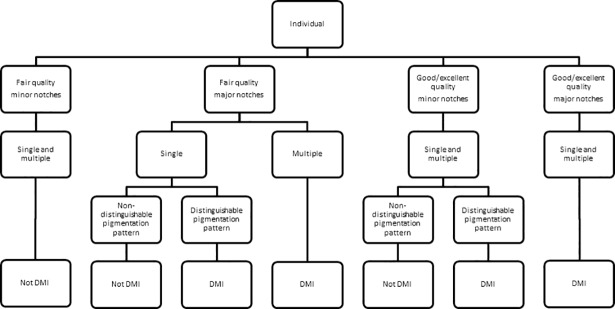
Distinctively marked individual (DMI) flow chart used to determine whether an individual adult common dolphin (*Delphinus* sp.) was distinctively marked and could be included in the Hauraki Gulf Common Dolphin Catalogue (HGCDC).

A threshold for distinctiveness was developed to ensure that individual dolphins were distinctive enough to be included in a MRC analysis. Such dolphins were referred to as distinctively marked individuals (DMIs) [4]. Distinctiveness was based on PQ, nick/notch size, the number of nicks and notches, and the presence/absence of a distinguishable pigmentation pattern (Fig 3). Here, a distinguishable pigmentation pattern was classified as the left side of a dorsal fin exhibiting contrasting grey or white patterns (e.g. edge outlines and/or clusters of pigment), that allowed observers to identify distinctive corresponding sections of pigmentation between individuals (Fig 4). A flow chart was subsequently used to determine if an individual was a DMI (Fig 5). Only individuals that were considered DMIs were integrated into the Hauraki Gulf Common Dolphin Catalogue (HGCDC).

#### The Hauraki Gulf Common Dolphin Catalogue (HGCDC)

The HGCDC is a curated collection of 2,083 individually identified common dolphin photographs, collected between 2010 and 2013, in a single reconciled database. Each new prospective individual was carefully examined and all matches scrutinized by at least two independent experienced observers. A unique identification number was only assigned when both observers independently found no matches within the existing catalogue. Only DMIs were included in the HGCDC. The catalogue contains the best image of the left side of each unique individual’s dorsal fin (referred to as the ‘best images’) and a database of the best image from each day an individual was observed (referred to as the ‘sightings database’).

All catalogued individuals were cross-matched by multiple researchers to reduce the likelihood of false-positive or false-negative errors. Dorsal fin pigmentation patterns were used as an independent secondary feature to aid in recognizing unique individuals, and to evaluate potential mark loss. In an effort to eliminate false-positive matches, the entire catalogue was extensively reviewed by five independent experienced researchers to ensure that all images with the same identification number were in fact the same individual. Moreover, a blind check, that consisted of matching 20% of the catalogue (*n* = 416), was conducted to estimate potential false-positive or false-negative errors. The rate of mark change was assessed for all individuals sighted more than once to evaluate the stability of nicks or notches over time.

### Data analysis

#### Mark-recapture

A ‘capture’ refers to a photographed DMI and includes its associated sighting data (e.g. date, time, and GPS position). For MRC analyses, the sighting records of dolphins captured during each sampling period were collated into a matrix of capture histories. Within the matrix, each dolphin was recorded as either not captured ‘0’ or captured ‘1’ within a given day (the sampling period). Data were further stratified into D1 individuals only and D1 & D2 individuals combined to estimate population parameters. To reduce potential sparseness while obtaining the most reasonable sampling interval [24], data were pooled by austral seasons: summer (December to February); autumn (March to May); winter (June to August), and spring (September to November). A discovery curve was plotted to identify the number of newly identified adult common dolphins within the study period. It must be noted that this discovery curve accounts for additions (immigration and births), but does not account for subtractions from the population (emigration or deaths).

#### Mark ratio: Proportion of marked/unmarked dolphins

To estimate total abundance, estimates were adjusted to account for the unmarked proportion of the population [55–56]. These proportions were calculated using two independent mark ratios for D1 (mark ratio 1; MR1) and D1 & D2 (mark ratio 2; MR2) individuals [23].

MR1 (${\hat{\theta }}_{1}$) was calculated for groups where not all individuals were captured (NAC) using the following formulas:

$${\hat{\theta }}_{1_{D1}}=\frac{number\;of\;high\;quality\;images\;with\;D1\;fins}{total\;number\;of\;high\;quality\;images\;with\;D1+D2+D3\;fins}$$

$${\hat{\theta }}_{1_{D1+D2}}=\frac{number\;of\;high\;quality\;images\;with\;D1+D2\;fins}{total\;number\;of\;high\;quality\;images\;with\;D1+D2+D3\;fins}$$

MR2 (${\hat{\theta }}_{2}$) was calculated for groups where all individuals were AC. Unlike MR1, this ratio was calculated based on the knowledge of group size together with the number of D1 individuals in each group using the following formulas:

$${\hat{\theta }}_{2_{D1}}=\frac{number\;of\;D1\;individuals\;in\;each\;group}{total\;group\;size}$$

$${\hat{\theta }}_{2_{D1+D2}}=\frac{number\;of\;D1+D2\;individuals\;in\;each\;group}{total\;group\;size}$$

Standard errors for both mark ratio estimates were calculated using the following formula [23]:

$$SE(\hat{\theta })=\sqrt{\frac{\hat{\theta }(1-\hat{\theta })}{n}}$$

where *n* is the sample size in each equation. The sample size for ${\hat{\theta }}_{1}$ was derived from the total number of high-quality photographs (images with individuals classified as D1, D2, and D3) in NAC groups. The sample size for ${\hat{\theta }}_{2}$ consisted of the total number of groups encountered.

A Z-test [102] was used to assess whether there was a significant difference between mark ratios.

#### Validation of model assumptions

The estimation of demographic parameters under Jolly-Seber MRC models requires several assumptions [24, 103]; the violation of these can lead to bias in population parameter estimates (Table 2). Where potential violations may have occurred, we outlined the measures taken to eliminate these (Table 2).

**Table 2:** Assessment of the assumptions for Jolly-Seber models MRC including the potential biases that may occur in population parameter estimates and the methods used to account for potential violations. Abbreviations: Goodness of fit (GOF). This table has been adapted from Parra et al. [59].

Assumption	Potential bias in estimates	Method(s) used to eliminate potential violations	References
Instantaneous sampling	Up	- Collecting captures from short-term photo-identification event - Sampling occasions were relatively short in duration (3 years) compared to the dolphins lifespan (decades)	[51]
Marks are not lost or missed	Up	- Regular sampling over three years - Collecting captures from short-term photo-identification events - Photographed only the left side of the dorsal fin - The use of pigmentation as a secondary identifying feature - Only used high quality images of highly distinctive and distinctive individuals - Application of a distinctivness threshold - Cross-matched and reviewed catalogued individuals - Blind error check on 20% of catalogued individuals	[51, 58]
Homogeneous survival	Down	- Only included adults - Ran GOF tests 3.SM and 3.SR - Excluded first capture of each individual	[51, 110]
Homogeneous capturability	Individual response: down Behavioural response: trap shy = up, trap happy = down	- Ran GOF test 2.CL and 2.CT - Constraint added to the first and last two capture probabilities - Encounter duration added as a covariate	[24, 51, 113]

#### Goodness of fit tests

Goodness of fit (GOF) tests were conducted to evaluate if model assumptions [24, 103] were fulfilled. GOF tests (test 2.CL, 2.CT, 3.SM, and 3.SR) using capture probabilities pooled by season and year were based on the fully parameterised Cormack-Jolly-Seber model and were run in U-CARE version 2.02 [104]. Test 2 evaluates the assumption that capture probabilities do not differ among individuals (heterogeneity). Test 2.CL determines whether there is variation in the time between re-encounters for captured and uncaptured individuals among sampling occasions (a significant result, trap effect lasts for more than one sampling interval) [104], and test 2.CT examines whether there is a behavioural response to capture (trap-shy statistic z<0, trap-happy statistic z>0) [104]. Test 3 evaluates the assumption that all identifiable dolphins have the same probability of survival between sampling occasions. In open models, mortality and emigration are confounded parameters; therefore, estimates of survival are in fact of ‘apparent survival’ [24]. Test 3.SM examines the effect of capture on apparent survival [104] and test 3.SR incorporates a statistic for transience (a significant result, z>0, P<0.05, individuals only observed once) [104]. To further examine the transience, test 3.SR was run using all captures and after removing the first capture (i.e. excluding transients). The removal of first captures resulted in the exclusion of 1,698 D1 individuals and 1,449 D1 & D2 individuals.

#### Model selection

The Schwarz and Arnanson ‘super-population’ POPAN approach (hereafter referred to as POPAN) was implemented using MARK version 8.0 [105]. Open population models were chosen based on the evidence that common dolphins are part of an open and larger population that move between regions along the north-eastern coastline of the North Island [86]. It is also likely that there are additions (births/immigration) and deletions (deaths/emigration) over the course of the study. In this context, the Robust Design was considered as a potential method since it allows estimation of temporary emigration [106]; however, this model requires an *a-priori* sampling design, that could not be applied in this study due to the logistical constraints of the tourism platform.

#### POPAN super-population approach

The super-population approach is based on a re-parameterization of the Jolly-Seber model with an additional parameter, ${\hat{N}}_{Super}$, to denote the size of the ‘super-population’ [51, 107]. In this study, the ‘population’ included dolphins that inhabit the HG during any given season. The ‘super-population’, included dolphins that visited the HG from the north-eastern coastline of the North Island over the three year study period. The ‘super-population’ abundance estimate was not considered accurate due to the additions (births) and deletions (mortality) that would have occurred during this study, and therefore the seasonal abundance was reported instead.

POPAN was used to estimate seasonal apparent survival (*ø*), capture probability (*p*), probability of entry (*β*), and abundance (N).

Estimates of survival presented here are of ‘apparent survival’ since in open models, emigration is confounded with mortality (i.e. death + emigration) and therefore true survival cannot be estimated [108–109]. To estimate apparent survival, the first capture of each individual was excluded [6, 21, 110] to avoid overinflating mortality/emigration (i.e. exclusion of transient dolphins). The analysis also excluded non-adult individuals, that eliminated possible heterogeneity arising from differences in age class.Given the gregarious nature of *Delphinus*, there was concern that encounter duration could also have an effect on capture probability. This is because limited encounter durations may reduce the probability of photographing all individuals within a group. To test for this, encounter duration was included as a covariate in the design matrix to evaluate whether it had an effect on capture probability.

The full set of candidate POPAN models were analysed (applying all possible combinations of parameter specifications). These included both time-dependent (t) and constant (.) apparent survival, capture probability and probability of entry. All models were run using excluding the first capture of each individual to account for the effect of transience (Test 3.SR). A additional model was run adding a constraint to the first and last capture probabilities to provide parameter identifiability for models with time variant survival and probability of entry [24].

Potential over-dispersion was examined by estimating the median variance inflation factor ($\hat{c}$). When $\hat{c}>1$, $\hat{c}$ was incorporated to produce a Quasi-like Akaike Information Criterion (QAICc) statistic, instead of an Akaike Information Criterion statistic [111]. The model with the lowest QAICc value was chosen as the most parsimonious model.

#### Abundance

The total abundance of all individuals (marked and unmarked) identified during the study period (${\hat{N}}_{Total}$) was calculated for both D1 (${\hat{N}}_{{Total}_{D1}}$) and D1 & D2 (${\hat{N}}_{{Total}_{D1+D2}}$) individuals [59] as follows:

$${\hat{N}}_{Total=\frac{{\hat{N}}_{m}}{\hat{\theta }}}$$

where ${\hat{N}}_{m}$ is the abundance estimate of marked individuals and $\hat{\theta }$ is the estimated proportion of marked individuals.

$\hat{\theta }$ was calculated for both D1 and D1 & D2 individuals (S2 Table). Here, the average $\hat{\theta }$ was calculated for each season to generate seasonal abundance estimates.

The variance of (${\hat{N}}_{Total}$) was derived [57–58] using the following formula:

$$SE({\hat{N}}_{Total})=\sqrt{{{\hat{N}}_{Total}}^{2}(\frac{{SE({\hat{N}}_{m})}^{2}}{{{\hat{N}}_{m}}^{2}}+\frac{1-\hat{\theta }}{n\hat{\theta }})}$$

where *n* included the number of high-quality photographs (D1, D2, and D3) in NAC groups.

Here *n* was calculated for each season for both D1 and D1 & D2 individuals (S2 Table).

Log-normal 95% confidence intervals (CI) were calculated [112] as follows:

$$C=exp\left(1.96\sqrt{ln(1+{\left(\frac{SE({\hat{N}}_{Total})}{{\hat{N}}_{Total}}\right)}^{2})}\right)$$

where the lower limit (${\hat{N}}_{Lower}$) was calculated as ${\hat{N}}_{Lower}={\hat{N}}_{Total}/C$ and the upper limit (${\hat{N}}_{Higher}$) was calculated as ${\hat{N}}_{Higher}={\hat{N}}_{Total}\times C$

## Results

### Photo-identification

Both yearly and seasonal effort increased throughout the study period (S4 Fig). A total of 419 photo-id surveys including 2,518 hours of survey effort were undertaken in the HG between January 2010 and December 2013 (Table 3). During these surveys, over 240,000 images were collected, of which 30,842 were deemed sufficient quality to detect DMIs. From this, a total of 2,083 unique individuals were identified within 1,411 groups (Table 3).

**Table 3:** Total seasonal photo-identification effort for adult common dolphins (*Delphinus* sp.) between 2010 and 2013 in the Hauraki Gulf, New Zealand (Individuals identified are the number of unique animals first sighted per season, whereas D1 and D2 represents the number of distinctive animals per category (D1 and D2) sighted and re-sighted per season). Abbreviations: Hours (h), summer (S), autumn (A), winter (W), and spring (Sp.). Survey duration refers to the total time on the water per survey (including time spent during encounters and searching for dolphins). Encounter duration refers to the total time during photo-id sessions with groups of dolphins. Search duration refers to the total time searching for dolphins where no photo-id sessions occurred.

***Year***	2010				2011				2012				2013					Total
*Season*	S	A	W	Sp.	S	A	W	Sp.	S	A	W	Sp.	S	A	W	Sp.	S	
*Survey duration (h)*	123.8	59.2	75.1	66.8	46.4	58.8	108.8	204.9	223.3	155.6	220.0	133.8	283.0	230.0	223.5	227.6	77.3	**2,518**
*Encounter duration (h)*	9.1	4.3	8.9	3.2	2.0	5.0	11.1	35.0	27.5	32.7	46.4	35.8	74.0	50.6	65.0	90.9	18.7	**520**
*Search duration (h)*	114.7	54.9	66.2	63.6	44.4	53.8	97.7	169.9	195.8	122.9	173.6	98	209	179.4	158.5	136.7	58.6	**1,998**
*Photo-id surveys (d)*	15	8	10	8	6	7	14	35	39	32	40	28	40	46	44	31	16	**419**
*Groups encountered*	23	13	24	25	8	12	50	128	83	102	145	101	166	123	163	191	54	**1,411**
*Sightings*	35	15	58	32	13	18	49	217	171	223	286	169	493	355	421	717	150	**3,422**
*Individuals identified*	33	13	58	30	11	18	45	182	110	141	180	106	258	181	240	405	72	**2,083**
*D1*	17	6	33	27	25	14	31	135	111	120	139	88	309	198	219	410	91	**1,973**
*D2*	14	7	24	18	4	4	18	78	55	93	140	81	173	149	196	293	58	**1,405**

PQ was classified as fair for 2.2% (*n* = 46), good for 59.1% (*n* = 1,232), and excellent for 38.7% (*n* = 805) of individuals. In addition, 51.3% (*n* = 1,069) and 48.7% (*n* = 1,014) of individuals were catalogued as D1 or D2, respectively. The number of individuals only sighted once was 66.2% (*n* = 1,379). A total of 33.8% of dolphins were photographed on more than one occasion (*n* = 704), up to a maximum of 15 times. On average, common dolphins were re-sighted on 1.7 (SE = 0.429) occasions between 2010 and 2013.

The discovery curve displayed a rapid, consistent inclinein the number of individuals identified, that continued until the end of the study period (December 2013; S5 Fig). This suggests that not all individuals were captured, and that common dolphins in the HG form part of an open population that has not been captured entirely. The number of individuals identified increased between mid-2012 to the end of 2013 (S5 Fig).

### Mark change and estimate of cataloguing error rate

The 704 individuals that were sighted more than once were assessed for mark change by inspecting images of the same dolphins in the order they were sighted. Of these, 16.1% changed over time. Mark changes included the addition of new nicks and notches as well as changes in original nick/notch size. Despite this, changes in marks were easily recognised because most individuals (95.3%) displayed pigmentation patterns that could be used as an independent secondary feature to aid identification [98], reducing the potential for mark loss or misidentification.

Aditionally, an examination of the cataloguing error rate was undertaken for 20% of the HGCDC (*n* = 416). This revision revealed 1 false-positive and 1 false-negative error, resulting in an error rate of 0.48%.

### Mark ratio

Of the 240,000 photographs of adult common dolphins encountered within all groups, 87.2% (*n* = 26,902) originated from NAC groups. From these photographs, 26.3% (*n* = 7,075) and 46.4% (*n* = 12,483) were classified as D1 and D1 & D2 individuals, respectively. Therefore, MR1 was 26.3% (SE = 0.003) for D1 and 46.4% (SE = 0.003) for D1 & D2 individuals.

A total of 71 groups were encountered where all individuals were captured (AC; 6.2%, *n* = 1,144). Within these groups, 766 individualswere photo-identified. Of these, 26.2% (*n* = 201) were D1 and 47.4% (*n* = 363) were D1 & D2 individuals. Thus, MR2 was 26.2% (SE = 0.013) for D1 and 47.2% (SE = 0.015) for D1 & D2 individuals, respectively.

No significant difference between ${\hat{\theta }}_{1_{D1}}$ and ${\hat{\theta }}_{2_{D1}}$ (p = 0.970), or between ${\hat{\theta }}_{1_{D1+D2}}$ and ${\hat{\theta }}_{2_{D1+D2}}$ (p = 0.552) were detected. As a result ${\hat{\theta }}_{1_{D1}}$ and ${\hat{\theta }}_{1_{D1+D2}}$ were used in all subsequentestimates.

### Goodness of fit tests

The global tests for D1 and D1 & D2 individuals were significant (p<0.001). There was no time variation between re-encounters (Test 2.CL) for D1 individuals; however, some variation was observed for D1 & D2 individuals (S3 Table). This may be the result of the large number of individuals identified with few recaptures. There was evidence of a behavioural response to capture for both D1 and D1 & D2 individuals (Test 2.CT; trap-shy’; S3 Table), that may have been caused by individual variation to boat approaches (i.e. some dolphins avoiding the vessels, [51]). However, the more likely explanation of this result isdue to the inability to capture all individuals during a sampling period (e.g. due to transiency), and/or the inability to complete homogenous surveys across the entire study area. It is possible that dolphins were present in other areas that were not sampled. Capture did not have an effect on apparent survival (Test 3.SM; S3 Table); however, we detected transiency (Test 3.SR; S3 Table). Due to the significant result of test 3.SR (biased downwards) we removed the first capture of each individual which resulted in a non-significant result for test 3.SR for D1 individuals and D1 & D2 individuals. We acknowledge that transiency may be an artefact of our sampling design confounded with ‘true’ transiency (animals that visit the area only once). It is possible that dolphins were present but were not photographed and/or were not in the area surveyed.

### POPAN models

#### D1 individuals

POPAN models were adjusted for an estimated median $\hat{c}$ = 1.14 (SE = 0.005). The model that best explained the data (ø_(.)_ p_(t)_ β_(.)_) incorporated constant apparent survival, time-varying capture probability, and constant probability of entry (Table 4). This model received 97.3% of the model weights.

**Table 4:** Top models from QAICc based-averagingfor sightings data of adult common dolphins (*Delphinus* sp.) photo-identified between January 2010 and December 2013 in the Hauraki Gulf, New Zealand. Model results are for POPAN data pooled by seasons and excluded the first capture of each individual. Models are shown for highly distinctive (D1) and highly distinctive and distinctive (D1 & D2) individuals. The lowest QAICc value represents the model that has the most support from the data (**in bold**). Abbreviations: Apparent survival (*ø*), capture probability (*p*), probability of entry (*β*), constant parameter (.), time-varying parameter (t), model likelihood (ML), and number of parameters (NP).

Nick/notch distinctiveness	Model number	Model	QAICc	Delta QAICc	QAICc Weights	ML	NP	Deviation
*D1*	***1***	***ø***_**(.)**_ ***p***_**(t)**_ ***β***_**(.)**_	**3410.77**	**0.000**	**0.973**	**1.000**	**20**	**-3929.03**
	*2*	*ø*_(.)_ *p*_(EnDur+t)_ *β*_(t)_	3419.15	8.38	0.015	0.015	36	-3953.75
	*3*	*ø*_(.)_ *p*_(t)_ *β*_(t)_	3419.60	8.83	0.012	0.012	35	-3951.21
	*4*	*ø*_(t)_ *p*_(t, t1 = t2, t15 = t16)_ *β*_(t)_	3448.99	38.22	0.000	0.000	48	-3949.15
	*5*	*ø*_(.)_ *p*_(.)_ *β*_(t)_	3692.24	281.47	0.000	0.000	19	-3645.51
	*6*	*ø*_(.)_ *p*_(.)_ *β*_(.)_	3882.45	471.68	0.000	0.000	4	-3424.87
*D1 & D2*	***7***	***ø***_**(.)**_ ***p***_**(t)**_ ***β***_**(t)**_	**4849.58**	**0.000**	**0.974**	**1.000**	**35**	**-7163.47**
	*8*	*ø*_(.)_ *p*_(t)_ *β*_(.)_	4857.10	7.52	0.023	0.023	20	-7125.38
	*9*	*ø*_(t)_ *p*_(t, t1 = t2, t15 = t16)_ *β*_(t)_	4870.32	20.68	0.000	0.000	48	-7169.60
	*10*	*ø*_(.)_ *p*_(EnDur+t)_ *β*_(t)_	4913.27	63.68	0.000	0.000	36	-7101.83
	*11*	*ø*_(.)_ *p*_(.)_ *β*_(t)_	5245.66	396.08	0.000	0.000	11	-6718.63
	*12*	*ø*_(.)_ *p*_(.)_ *β*_(.)_	5619.86	770.28	0.000	0.000	4	-6330.36

#### D1 & D2 individuals

POPAN models were adjusted for an estimated median $\hat{c}$ = 1.28 (SE = 0.005). The model that best explained the data (ø_(.)_ p_(t)_ β_(t)_) incorporated constant apparent survival, time-varying capture probabilities, and time-varying probability of entry (Table 4). This model received 97.4% of the model weights.

### Population parameters: apparent survival, capture probability, probability of entry, and abundance

The best model resulted in an estimate of constant apparent survival of 0.767 (CI = 0.694–0.827) for D1 individuals and 0.796 (CI = 0.729–0.850) for D1 & D2 individuals.

Estimates of capture probability varied over time for D1 individuals. The lowest estimate was 0.021 (CI = 0.011–0.041) in summer 2010–2011, while the highest estimate was 0.283 (CI = 0.244–0.326) in spring 2013. Similar variability in estimates of capture probability were also detected for D1 & D2 individuals. Capture probability ranged from 0.006 (CI = 0.003–0.012) in summer 2010–2011 to 0.199 (CI = 0.167–0.235) in summer 2012–2013.

The probability of entry remained constant at 0.062 (CI = 0.062–0.062) for D1 individuals. However, for D1 & D2 individuals, the probability of entry varied seasonally from 0.000 (CI = 0.000–0.866) in autumn 2012 to 0.413 (CI = 0.313–0.521) in winter 2010.

**Fig 6:**
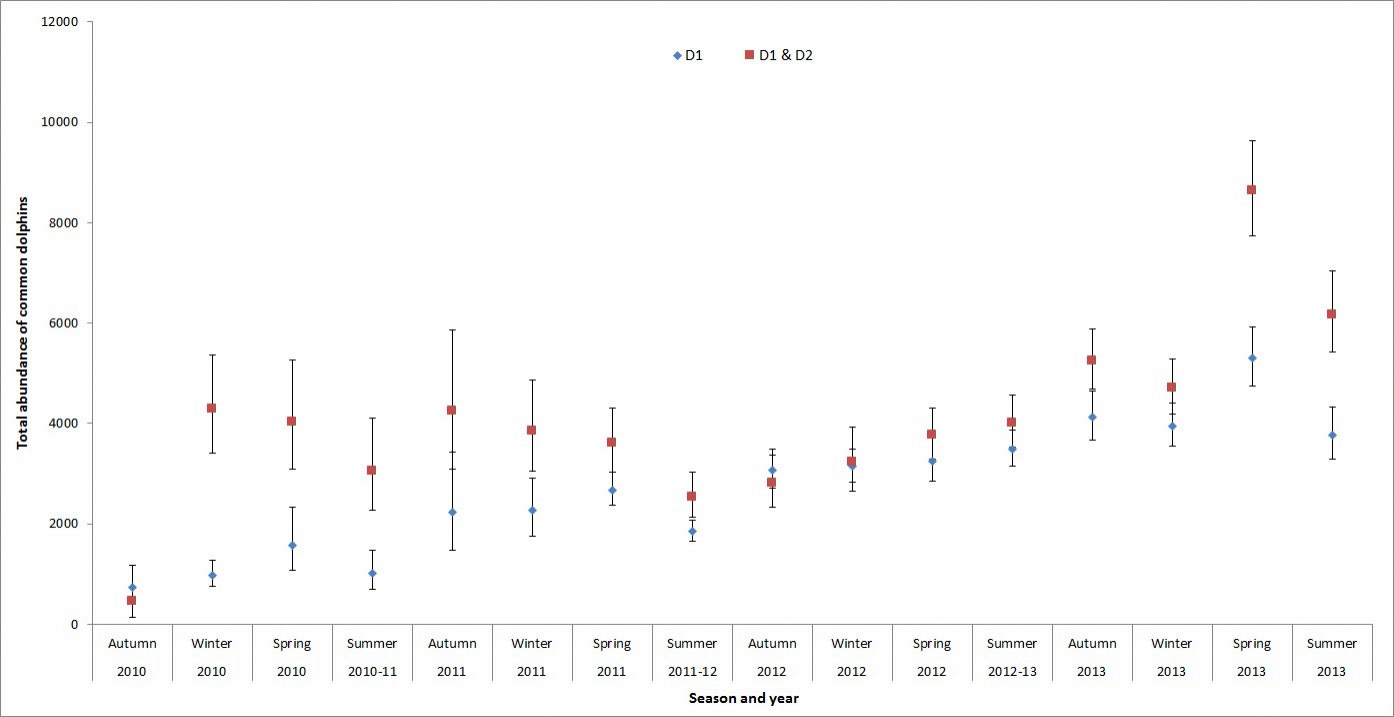
Seasonal abundance estimates (±CI) for adult (marked and unmarked) common dolphins (*Delphinus* sp.) between January 2010 and December 2013 in the Hauraki Gulf, New Zealand, obtained using POPAN. Estimates are given for highly distinctive (D1) and highly distinctive and distinctive (D1 & D2) individuals.

For D1 individuals, seasonal abundance of both marked and unmarked dolphins varied from 732 (CI = 460–1,177) in autumn 2010 to 5,304 (CI = 4,745–5,930) in spring 2013 (Fig 6). The seasonal estimates for D1 & D2 individuals ranged from 465 (CI = 148–1,488) in autumn 2010 to 8,632 (CI = 7,738–9,630) in spring 2013 (Fig 6).

## Discussion

Knowledge of population parameters is important to allow detection of population changes over time [51]. However, abundance is rarely estimated for poorly marked gregarious species because of the inherent challenges of studying them. Despite this, the present study estimated that seasonal abundance estimates for adult common dolphins in the HG (D1 & D2 individuals) ranged from 465 (CI = 148–1,488) in autumn 2010 to 8,632 (CI = 7,738–9,630) in spring 2013. This supports earlier suggestions that this area represents an important region for common dolphins within New Zealand waters [114]. Previous studies have identified that common dolphins in the HG occur year round [94, 114–115], in contrast to other regions around New Zealand [116–119]. Furthermore, it has been suggested that this region is also fundamental for feeding and nursing [29, 114]. The estimated abundance of common dolphins in the HG is therefore not surprising considering these animals appear to use these waters with purpose and regularity [29, 114].

### Challenges of MRC analysis for gregarious poorly marked species

#### Unequal coverage of the study area

Applying photo-id and MRC analysis to poorly marked gregarious species presented a number of challenges. One difficulty was the inability to cover the study area homogeneously. Due to the large size of the inner HG and notably, the logistical constraints of the tourism platform, systematic surveys were considered unfeasible for conducting photo-id within this region. Non-systematic surveys were instead conducted, but not without their limitations. For example, non-systematic sampling prevented the use any models that assumed demographic and geographic closure. In addition, the changing spatial extent and allocation of effort meant that our surveys may have encountered animals that were not surveyed or encountered in additional years. This may have artificially depressed estimates of apparent survival and capture probability. Our best effort to sample this large region and identify as many individuals as possible was to combine data from both the tourism and research vessels to enable extended spatial coverage of the broader inner HG waters [31, 95, 120].

#### Mark ratio and individual distinctiveness

The inclusion of both D1 and D1 & D2 individuals into MRC analysis proved to be a useful approach to increase the number of animals included in the analyses, that resulted in higher capture rates over time. However, when dealing with poorly marked gregarious species, that have few re-sightings, a compromise is sometimes required between PQ and the number of photographs that are included in the MRC analysis. In this study, fair quality (in combination with good and excellent quality) photographs were only included for D1 (because other identifying features were deemed sufficient enough to aid in identification) but not for D2 individuals. This approach increased the number of photographs included in the MRC analysis increasing our ability to identify unique individuals.

Low levels of distinctiveness, as exhibited for common dolphins, can increase the number of false-positive and false-negative errors when cataloguing individuals [121]. Consequently, this can lead to a violation of the MRC assumption that marks are not lost or missed. In an effort to avoid this violation, only DMIs were included into MRC analysis. This study applied a structured threshold for distinctiveness to address the lack of independence between PQ and ND. While such measures may not be as important in studies of more distinctive species (such as Indo-Pacific bottlenose dolphins, *Tursiops aduncus*; 100% considered marked) [20], they are critical for individual identification of less distinctive animals. The threshold for distinctiveness implemented in the present study strengthened the reliability of identifying unique individuals, and therefore the robustness of MRC analysis. Individual distinctiveness was significantly improved by introducing pigmentation patterns as a secondary independent identification feature. While some studies have reported that pigmentation patterns are not temporally reliable [74], pigmentation patterns proved stable for over a decade incommon dolphins in this region [98], providing a useful independent secondary feature to uniquely identify individuals.

One advantage of only using D1 animals is that abundance estimates are more robust against the misidentification of animals. Alternatively, when using D2 individuals, misidentification of individuals may result in abundance estimates being under- or over-inflated [122]. We therefore recommend that D2 individuals are only included for abundance estimation when misidentification is unlikely and/or sufficient effort has been made to reduce the number of misidentifications. Here, misidentification was considered unlikely considering that 83.9% of individuals showed no change in markings over the four year study period. Furthermore, multiple identifying features were used to minimise the misidentification of animals. For example, pigmentation patterns were present for a majority of catalogued individuals (95.3%) [98], and aided as a secondary feature to reduce misidentifications. Likewise, using strict PQ and ND criteria, in combination with the use of a distinctiveness threshold, further minimised the misidentification of individuals. Moreover, the addition of D2 animals allowed a larger number of individuals to be included in the analysis.

#### Transiency

The level of true transiency within this open population may, be higher due to the wide-ranging nature of *Delphinus* and their sometimes large group sizes, that makes capturing all individuals difficult. Moreover, despite common dolphins within this region forming part of a larger open population, data collection was restricted to the inner HG that represents only a small proportion of their home range. Also, given the large number of dolphins within this region, the extent of true migration is likely to be underestimated. Transiency resulted in heterogeneous data, thereby violating the assumptions that all individuals have the same probability of survival between sampling occasions. To avoid underestimating apparent survival, the first capture of each individual was excluded (i.e. excluding transient animals) in [6, 21, 110]. Despite still indicating transience, the POPAN model structures were correct. Moreover, the $\hat{c}‑\mathrm{values}$ indicate that the excess variation was within acceptable limits [111]. It is also possible that some of the animals identified as transient within our study area may return at a later time or may permanently leave the region. Only future studies on individual movement patterns and subsequent long-term analysis of photo-identification datasets will be able to determine the fate of individuals.

#### Temporary emigration

Temporary emigration occurs when some animals are absent from the sampling site during one or several sampling occasions [123]. Examining temporary emigration rates (either markovian or random) allows modelling of heterogeneity in capture probability. Robust design models [105–106] have been used successfully to estimate temporary emigration and other population parameters in several species of delphinids [4, 6, 20, 66, 124]; however, this method requires an *a-priori* sampling design that allows planning for discrete sampling intervals between open primary periods while secondary periods are closed (demographic and geographic closure). In our study, these last two requirements could not be fulfilled given the open-nature of this population, the use of non-systematic surveys and the high number of dolphins sighted only once (66.2%), all of which would have deemed the closure assumption not possible.

### Population parameters

#### Apparent survival

Estimates of apparent survival for common dolphins in the HG were 0.77 and 0.80 for D1 and D1 & D2 individuals, respectively. To our knowledge, the only other survival estimate (0.40, SE = 0.110) available to date for common dolphins is for a mixed population of common and striped dolphins (*Stenella coeruleoalba*) in the Gulf of Corinth, Greece [81]. Unfortunately, comparisons of apparent survival between Bearzi et al. [81] and the present study are not comparable since the former examined a mixed species population. Comparisons can be made with other cetacean species. For example, annual estimates of apparent survival for spinner dolphins (*S*. *longirostris*) in Hawai`i, were 0.97 (SE±0.05) [23]. This survival estimate was high, presumably because the Hawai`i Island spinner dolphin stock may form part of a closed population [125]. In comparison, the low apparent survival estimates presented in our study may be due to high levels of temporary or permanent emigration; however, some levels of mortality cannot be discounted.

#### Capture probabilities

While no trend in capture probabilities was evident, variation did exist across years and seasons. Although such variation could be explained by changes in abundance and distribution of common dolphins in the HG, it is highly likely that variation in capture probabilities was caused in part by differences in sampling effort. Throughout the present study, there were a number of changes in sampling effort and design, which may have been reflected in variation in capture probabilities. For example, during the first six seasons of this study (autumn 2010 to winter 2011), photo-id was only conducted opportunistically. Likewise, total seasonal encounter duration (in hours, range = 2.0–11.1) and capture probabilities (range = 0.006–0.062) during this period were low compared with other seasons. However, in spring 2011 dedicated photo-id surveys began from a tourism platform. During this time, total seasonal encounter duration increased by 68.2% (from 11.1 hrs in winter to 34.9 hrs in spring). Similarly, capture probabilities increased by 76.5% (from 0.028 in winter to 0.117 in spring). Dedicated photo-id surveys then began from a research vessel in Summer 2012, increasing encounter durations and therefore the ability to capture more individuals. During this time, total seasonal encounter duration and capture probability increased to 74.0 hrs and 0.199, respectively. Such results indicate that differences in the nature of photo-id surveys (opportunistic versus dedicated), the type of vessel used (tourism platform or research vessel), and the time spent with animals (total seasonal encounter duration) can have a significant effect on the capture probabilities presented in MRC models. Similar relationships between sampling effort and capture probabilities have been reported [21], highlighting the impact of survey design on estimates of capture probabilities. In addition, the number of researchers conducting photo-id was not standardised across the survey period that may have resulted in heterogeneity of capture probabilities between surveys. Considering the variations described here, the observed increase in capture probabilities over time may be more reflective of methodologies employed throughout this study rather than true ecological patterns. Future studies should aim to standardise methods used across entire survey periods to mitigate this issue.

#### Abundance

Despite *Delphinus* being widely distributed [126], there is a paucity of abundance estimates for this genus [82]. Most published MRC studies for *Delphinus* have focused on establishing catalogues of known individuals, although abundance estimates were not generated [79–80, 86]. Worldwide, there are only two published reports of common dolphin abundance using MRC methods [80–81], both that were for small populations (15 and 28 individuals, respectively). This study presents the first abundance estimate using MRC methods based on a large catalogue (≥500 individuals) of common dolphins.

Different levels of ND affected estimates of abundance. For example, when only including D1 individuals, seasonal abundance varied from 732 (CI = 460–1,177) in autumn 2010 to 5,304 (CI = 4,745–5,930) in spring 2013. However, when both D1 and D2 categories were included, seasonal estimates ranged from 465 (CI = 148–1,488) in autumn 2010 to 8,632 (CI = 7,738–9,630) in spring 2013. This result was unexpected as the mark ratio adjustments should have accounted for differences in the level of ND included for each estimate. One explanation for this result is that the best model selected for D1 individuals included constant survival and probability of entry as well as time dependant capture probability (*ø*_(.)_ *p*_(t)_ *β*_(.)_), whereas for D1 & D2 individuals combined, probability of entry varied by time (*ø*_(.)_ *p*_(t)_ *β*_(t)_). Furthermore, it is possible that the mark ratio may have been underestimated for D1 individuals and/or overestimated for D1 & D2 individuals. Finally, the best model selected for D1 & D2 may reflect a larger number of animals entering the study area between sampling occasions. As this dataset includes more animals, this effect may be stronger than for the D1 only dataset and hence, picked up by the model selection process.

The proportion of marked animals can also affect the reliability of abundance estimates. For example, the mark ratio was 26.3% (SE = 0.003) for D1 individuals and 46.4% (SE = 0.003) for D1 & D2 individuals. When comparing abundance estimates for autumn 2012, 3,078 (CI = 2,709–3,498) individuals were reported for D1 whereas 2,804 (CI = 2,399–3,360) were reported for D1 & D2 individuals. This illustrates that the abundance of D1 & D2 individuals is lower in autumn 2012 because the proportion of unmarked animals is also lower for D1 & D2 individuals (53.6% unmarked) when compared with D1 individuals alone (73.7% unmarked). This result suggests that estimates for D1 individuals may be overestimated, and therefore the inclusion of D2 individuals likely generated more accurate abundance estimates (due to the larger number of individuals/re-sightings analysed).

Abundance estimates presented here do not exhibit seasonality, a result that is consistent with previous studies indicating year round occurrence [95, 115]. Common dolphins have been reported to occur in the HG in large aggregations (>50 animals) more frequently than expected during winter and spring [95], however this variation was not observed here. While seasonal abundance estimates showed a slightly upwards trend, this may have been caused by the increasing photo-id effort throughout the duration of the study. In addition, variation in seasonal abundance is also likely to have been influenced in part by differences in sampling effort over the four-year study period.

This study demonstrates that abundance can be estimated for both D1 individuals and D1 & D2 individuals combined. Seasonal abundance estimates were, however, different for D1 individuals when compared with D1 & D2 combined, which indicated that one, or the other, or both could potentially present some biases. This is a complex issue since bias could occur for both estimates in the calculation of mark ratio, ${\hat{N}}_{m}$ and/or ${\hat{N}}_{Total}$, and could be either a positive or negative bias. As previously stated, it is possible that the mark ratio may have been underestimated for D1 individuals and/or overestimated for D1 & D2 individuals. If the mark ratio was underestimated for D1 individuals, this would result in an over estimate of abundance. Despite our examination, the underlying factors associated with bias for both estimates remain unclear. Therefore, while the inclusion of D2 individuals may offer a more realistic estimate of abundance for the total population, the inability to determine the additional sources of bias means that the more conservative estimate of abundance (i.e. D1 only) should be adopted for management purposes.

## Conclusions

Conducting MRC analysis on poorly marked gregarious delphinids such as common dolphins presents a number of challenges, including the high portion of unmarked animals, low levels of distinctiveness, and the gregarious nature of *Delphinus*. The present study; however, illustrates that plausible estimates of apparent survival, capture probabilities, probability of entry, and abundance can be generated. This study presents the first abundance estimate using MRC methods for a large population of *Delphinus*, and demonstrates that such studies are possible for low marked gregarious species. A number of reliable photo-id protocols were useful for accounting for the low mark ratio for *Delphinus*. The combination of nicks and notches and dorsal fin pigmentation patterns provided a robust method for individual identification. Likewise, using strict PQ and ND criteria ensured that all individuals could be reliably identified. Identification was further assisted by the use of a distinctiveness threshold, that enabled PQ and ND to be assessed independently. Stratification of the data by distinctiveness was also a useful technique to identify the most accurate estimates of population parameters. In an effort to minimise potential violations to MRC assumptions we excluded transient animals from estimates of population parameters. We also added constraints/covariates when estimating capturability. Population parameters estimated within this study should be used for future monitoring of *Delphinus* populations on the New Zealand north-eastern coastline of the North Island and for similar poorly marked gregarious species of delphinids worldwide.

## Supporting information

S1 Fig Definitions of age-classes recorded for common dolphins (*Delphinus sp*.) in the Hauraki Gulf, New Zealand [1]. (DOCX)

S2 Fig Photographic quality (PQ) categories used to examine adult common dolphin images in the Hauraki Gulf, New Zealand. Images were classified as: a) poor; b) fair; c) good, or; d) excellent quality.
(DOCX)

S3 Fig Nick/notch depth categories used to examine adult common dolphin (*Delphinus* sp.) images in the Hauraki Gulf, New Zealand. Individuals were classified as having either minor or major nicks/notches [4].
(DOCX)

S4 Fig Survey tracks (black lines) of tour and research vessels for each year (a) and season (b) in the inner Hauraki Gulf, New Zealand.
(DOCX)

S5 Fig Cumulative discovery curve (black line) indicating the number of newly identified adult common dolphins (*Delphinus* sp.) between January 2010 and December 2013 in the Hauraki Gulf, New Zealand, including a 1:1 slope (grey line) as if all individuals documented were new additions to the catalogue. (DOCX)

S1 Table Description of attribute criteria used to examine the photographic quality (PQ) of common dolphin images in the Hauraki Gulf, New Zealand. Images were assessed according to focus, exposure, orientation, and visible percentage (adapted from [2–3]). When assessing quality criteria each attribute was considered independently to avoid bias/contradictions between categories being assessed.
(DOCX)

S2 TableSample sizes (*n*, the number of high-quality photographs for highly distinctive (D1), distinctive (D2) and non-distinctive (D3) individuals) and proportions of marked individuals ($\hat{\theta }$) used to estimate either the seasonal or super-population abundance of common dolphins using the Hauraki Gulf between 2010 and 2013.Here *n*_*Total*_ represents the sum of *n*_*D*1_, *n*_*D*2_, and *n*_*D*3_.(DOCX)

S3 TableResults of goodness of fit (GOF) tests conducted in U-CARE 2.02 in a Cormack-Jolly-Seber framework for adult common dolphins (*Delphinus* sp.) photo-identified between January 2010 and December 2013 in the Hauraki Gulf, New Zealand.Results are also included from the global test (GT; test 2+3). GOF tests were conducted for highly distinctive individuals (D1) only, and highly distinctive and distinctive individuals (D1 & D2) combined. Test 3.SR was re-run excluding the first capture of each individual and results are shown in italics. Values in bold indicate significance. Abbreviations: nick distinctiveness (ND), variance inflation factor ($\hat{c}$) and not applicable (na).(DOCX)
