# Correction: Challenges of implementing Mark-recapture studies on poorly marked gregarious delphinids

**DOI:** 10.1371/journal.pone.0203356

**Published:** 2018-08-28

**Authors:** Krista Hupman, Karen A. Stockin, Kenneth Pollock, Matthew D. M. Pawley, Sarah L. Dwyer, Catherine Lea, Gabriela Tezanos-Pinto

Affiliations 1 and 2 are listed out of order. The affiliations should appear as shown here: Krista Hupman^1,2^, Karen A Stockin^1^, Kenneth Pollock^3^, Matthew D. M. Pawley^1^, Sarah L. Dwyer^1^, Catherine Lea^1^, Gabriela Tezanos-Pinto^1^

**1** Coastal-Marine Research Group, Institute of Natural and Mathematical Sciences, Massey University, Auckland, New Zealand, **2** National Institute of Water and Atmospheric Research, 301 Evans Bay Parade, Wellington, 6021, New Zealand, **3** Department of Applied Ecology, North Carolina State University, Raleigh, North Carolina, United States of America
