# Supplementary material for: Challenges of implementing Mark-recapture studies on poorly marked gregarious delphinids
Source: PLoS One. 2018 Jul 11;13(7):e0198167. doi: 10.1371/journal.pone.0198167 (PMC6040702; doi:10.1371/journal.pone.0198167)
Supplement: S1 Fig — (DOCX) [file pone.0198167.s001.docx]

**S1 Fig**

| ***Age-class*** | **Definition** | **Example** |
| --- | --- | --- |
| *Immature (including neonates, calves and juveniles)* | Neonates: Small calves that exhibited diagnostic features indicative of newborns (e.g. the presence of dorso-ventral foetal folds, indicated here by red arrows). | 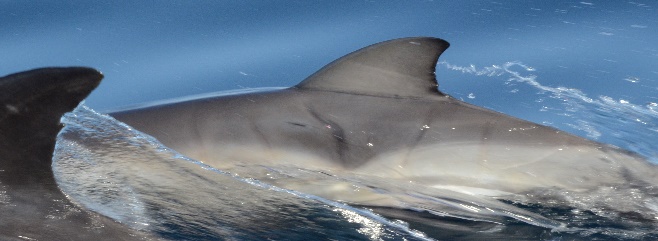 |
|  | Calves: Animals approximately ≤half the length of an adult and consistently observed in association with an adult animal. | 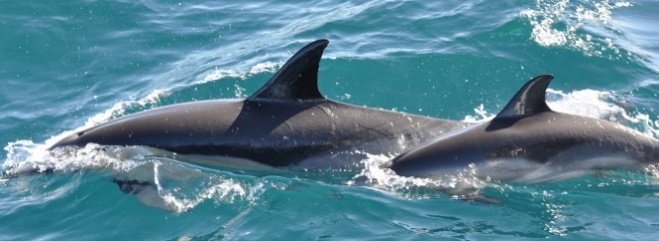 |
|  | Juveniles: Animals approximately two-thirds the size of an adult and frequently observed swimming in association with an adult animal but not in the infant position, suggesting that they were weaned. | 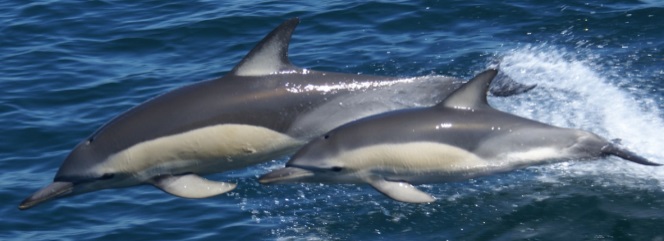 |
| *Adult* | All animals not included in the prior classifications. Adults reach up to approximately 1.8 m in length. | 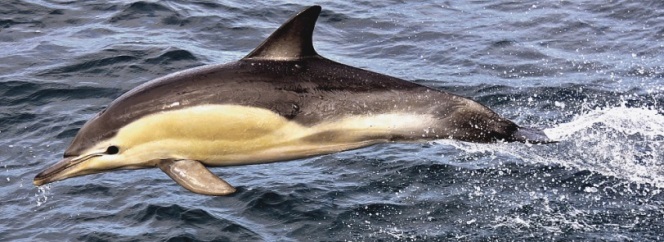 |

**Reference**

Stockin KA, Binedell V, Wiseman N, Brunton DH, Orams MB. Behavior of free-ranging common dolphins (*Delphinus* sp.) in the Hauraki Gulf, New Zealand. Mar. Mamm. Sci. 2009a;25: 283-301
