# Supplementary figures and images for: Challenges of implementing Mark-recapture studies on poorly marked gregarious delphinids
Source: PLoS One. 2018 Jul 11;13(7):e0198167. doi: 10.1371/journal.pone.0198167 (PMC6040702; doi:10.1371/journal.pone.0198167)

**S2 Fig**

| **PQ category** | **Score** | **Example** |
| --- | --- | --- |
| *Poor* | ≥11 | 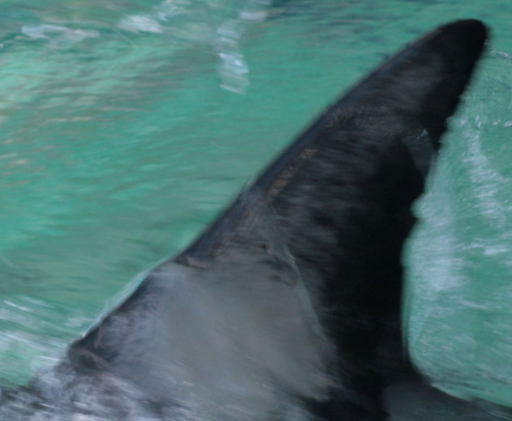 |
| *Fair* | 10 | 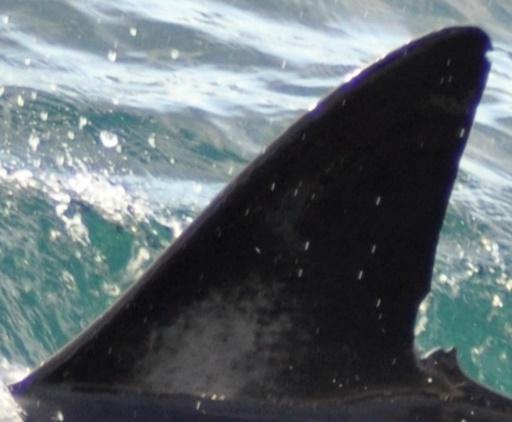 |
| *Good* | 7–9 | 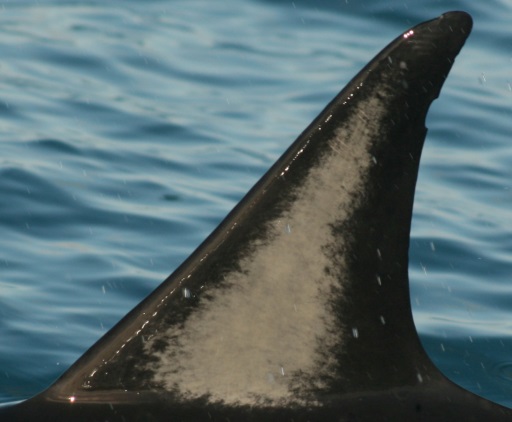 |
| *Excellent* | 4–6 | 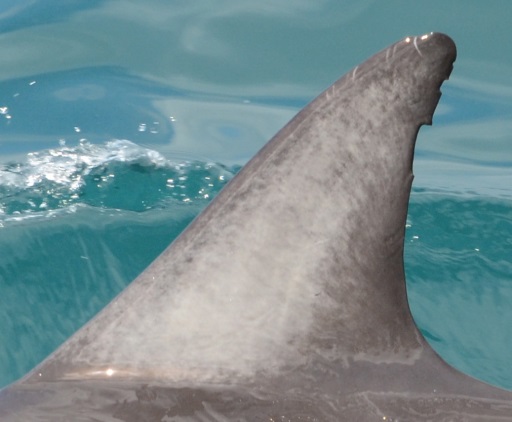 |

Supplement: S2 Fig — Images were classified as: a) poor; b) fair; c) good, or; d) excellent quality. (DOCX) [file pone.0198167.s002.docx]

**S4 Fig**

**(a) (b)**

**
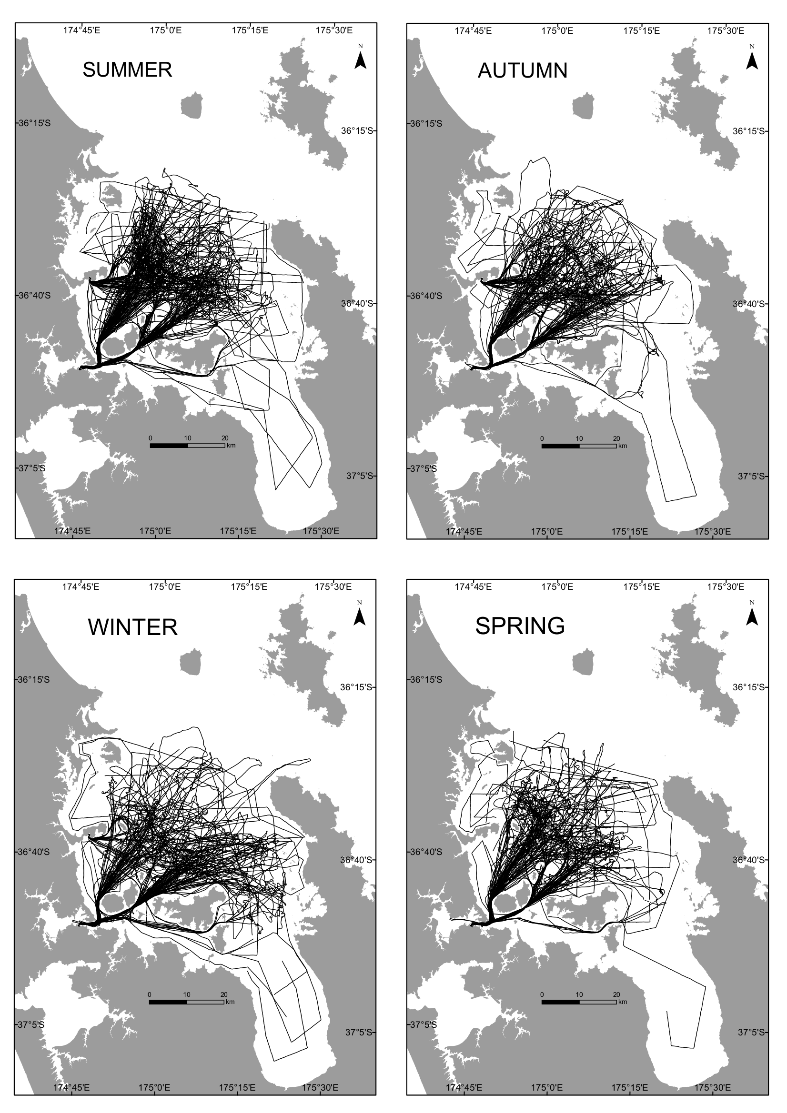

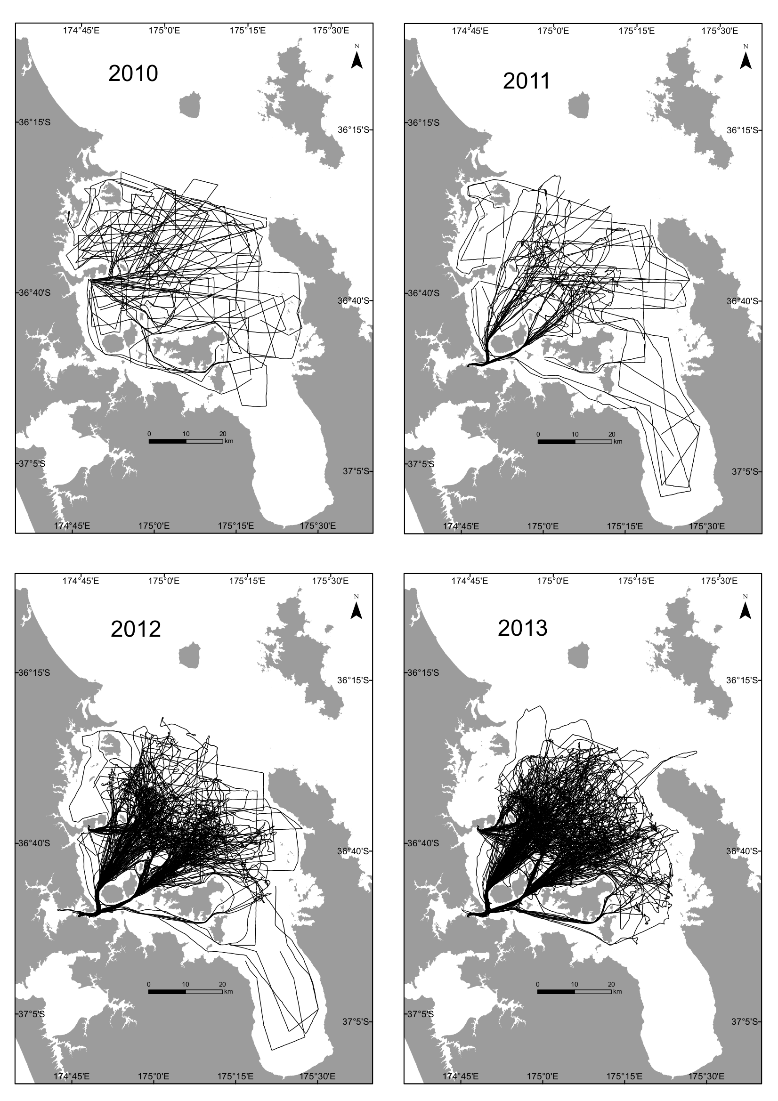
**

Supplement: S4 Fig — Survey tracks (black lines) of tour and research vessels for each year (a) and season (b) in the inner Hauraki Gulf, New Zealand. (DOCX) [file pone.0198167.s004.docx]

**S5 Fig**

**
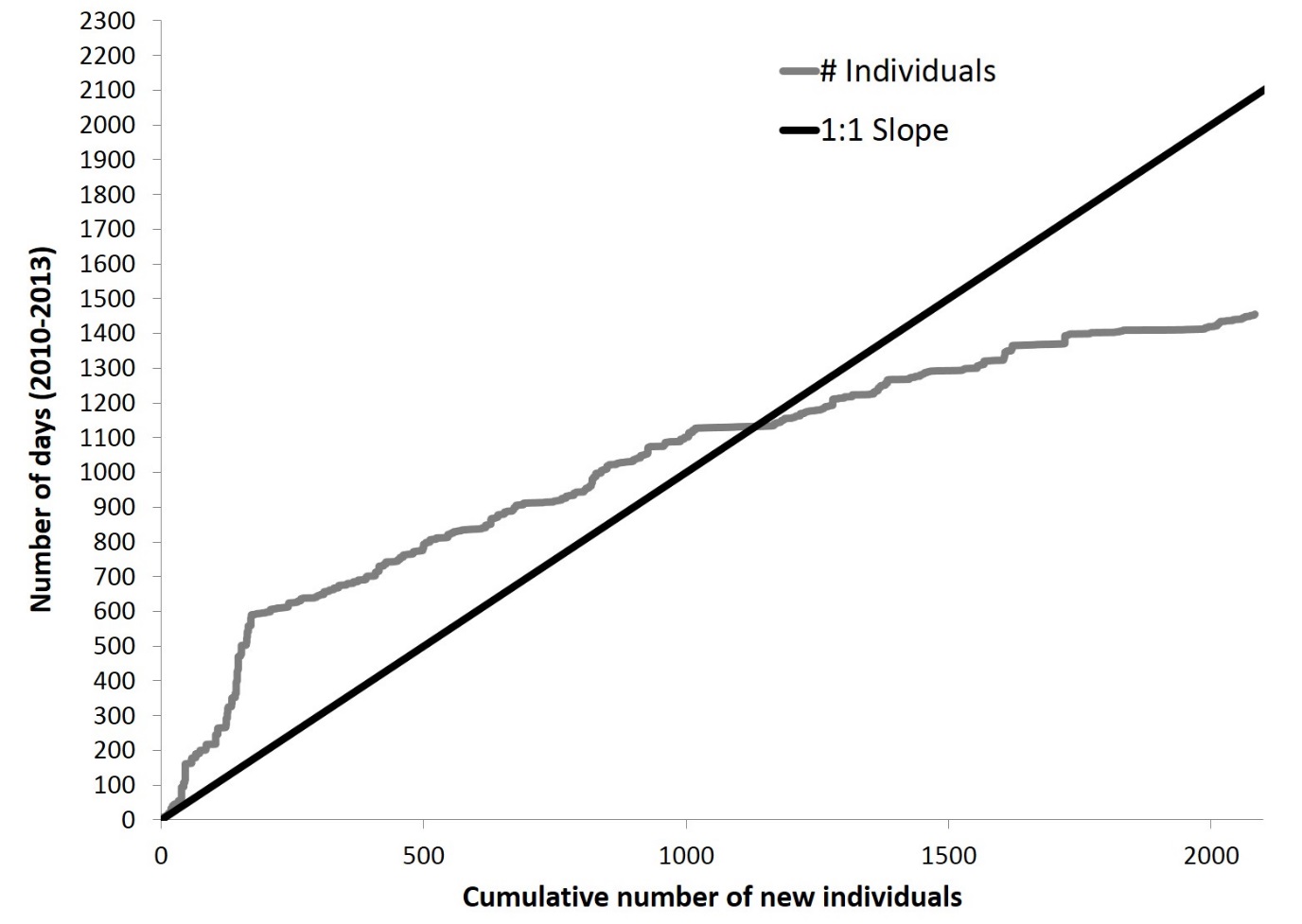
**

Supplement: S5 Fig — (DOCX) [file pone.0198167.s005.docx]
