# Supplementary material for: Challenges of implementing Mark-recapture studies on poorly marked gregarious delphinids
Source: PLoS One. 2018 Jul 11;13(7):e0198167. doi: 10.1371/journal.pone.0198167 (PMC6040702; doi:10.1371/journal.pone.0198167)
Supplement: S3 Fig — Individuals were classified as having either minor or major nicks/notches [4]. (DOCX) [file pone.0198167.s003.docx]

**S3 Fig**

| **Nick/notch category** | **Description** | **Example** |
| --- | --- | --- |
| *Minor* | Relative proportion of the depth of the nick/notch is <10% of the total base of the dorsal fin. Nick/notch is <1 cm in size. | 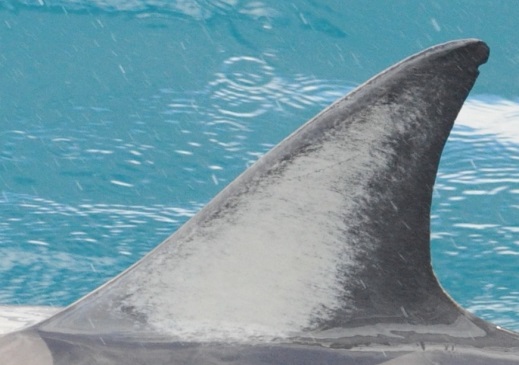 |
| *Major* | Relative proportion of the depth of the nick/notch is ≥10% of the total base of the dorsal fin. Nick/notch is ≥1 cm in size. | 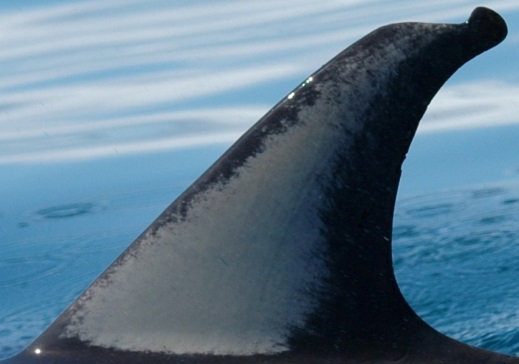 |

**Reference**

Luksenberg J. Prevalance of external injuries in small cetaceans in Aruban waters, southern Caribbean. PLoS ONE. 2014;9: e88988.
