# Supplementary material for: Challenges of implementing Mark-recapture studies on poorly marked gregarious delphinids
Source: PLoS One. 2018 Jul 11;13(7):e0198167. doi: 10.1371/journal.pone.0198167 (PMC6040702; doi:10.1371/journal.pone.0198167)
Supplement: S1 Table — Images were assessed according to focus, exposure, orientation, and visible percentage (adapted from [2–3]). When assessing quality criteria each attribute was considered independently to avoid bias/contradictions between categories being assessed. (DOCX) [file pone.0198167.s006.docx]

**S1 Table**

| *Attribute* | Description | Score |
| --- | --- | --- |
| *Focus* | - Poor: considerable blur - general outline and/or details are not visible - Reasonable: some blur - general outline visible and small nicks may not be entirely visible - Excellent: no blur - sharp outline and all details are visible | 9  4  1 |
| *Exposure (to light)* | - Poor: Under or over-exposed, only some details are seen - Reasonable: A little light or dark but all details are clearly seen - Excellent: No over or under exposure and all details and outline are visible | 9  3  1 |
| *Orientation* | - Poor: >45° to Perpendicular - Reasonable: ≤45° - Excellent: Parallel | 9  2  1 |
| *Percentage visible* | - Poor/reasonable: The leading and trailing edges of the dorsal fin are partially obscured - Excellent: The leading and trailing edges of the dorsal fin are fully visible | 8  1 |

**References**

Urian KW, Hohn AA, Hansen LJ. Status of the photo-identification catalog of coastal bottlenose dolphins of the western North Atlantic. Report of a workshop of catalogue contributors. National Oceanic and Atmospheric Administration Administrative Report, NMFS-SEFSC-425. North Carolina: National Oceanic and Atmospheric Administration; 1999. pp. 298-321.

Nicholson K, Bejder L, Allen SJ, Krützen M, Pollock KH. Abundance, survival and temporary emigration of bottlenose dolphins (*Tursiops* sp.) off Useless Loop in the western gulf of Shark Bay, Western Australia. Mar. Freshw. Res. 2012;63: 1059-1068.
