# Supplementary material for: Challenges of implementing Mark-recapture studies on poorly marked gregarious delphinids
Source: PLoS One. 2018 Jul 11;13(7):e0198167. doi: 10.1371/journal.pone.0198167 (PMC6040702; doi:10.1371/journal.pone.0198167)
Supplement: S2 Table — Here nTotal represents the sum of nD1, nD2, and nD3. (DOCX) [file pone.0198167.s007.docx]

**S2 Table**

| ***Abundance estimate*** | **Year** | **Season** | $\boldsymbol{n}_{\boldsymbol{D}\boldsymbol{1}}$ | $\boldsymbol{n}_{\boldsymbol{D}\boldsymbol{2}}$ | $\boldsymbol{n}_{\boldsymbol{D}\boldsymbol{3}}$ | $\boldsymbol{n}_{\boldsymbol{Total}}$ | ${\hat{\boldsymbol{\theta}}}_{\boldsymbol{1}_{\boldsymbol{D}\boldsymbol{1}}}$ | ${\hat{\boldsymbol{\theta}}}_{\boldsymbol{1}_{\boldsymbol{D}\boldsymbol{1\&D}\boldsymbol{2}}}$ |
| --- | --- | --- | --- | --- | --- | --- | --- | --- |
| *Seasonal* | 2010 | Autumn | 20 | 19 | 35 | 74 | 19.0 | 48.6 |
|  | 2010 | Winter | 67 | 39 | 63 | 169 | 25.9 | 51.1 |
|  | 2010 | Spring | 26 | 13 | 47 | 86 | 22.8 | 45.3 |
|  | 2010*–*11 | Summer | 18 | 2 | 15 | 35 | 45.2 | 64.0 |
|  | 2011 | Autumn | 19 | 16 | 30 | 65 | 24.7 | 43.3 |
|  | 2011 | Winter | 46 | 31 | 86 | 163 | 28.3 | 45.2 |
|  | 2011 | Spring | 285 | 200 | 504 | 989 | 27.0 | 45.5 |
|  | 2011*–*12 | Summer | 307 | 122 | 205 | 634 | 43.0 | 61.2 |
|  | 2012 | Autumn | 269 | 208 | 401 | 878 | 28.3 | 52.3 |
|  | 2012 | Winter | 532 | 558 | 699 | 1,789 | 29.8 | 56.5 |
|  | 2012 | Spring | 230 | 204 | 354 | 788 | 30.8 | 57.3 |
|  | 2012*–*13 | Summer | 1,095 | 644 | 1,385 | 3,124 | 30.4 | 50.8 |
|  | 2013 | Autumn | 517 | 564 | 802 | 1,883 | 27.0 | 53.5 |
|  | 2013 | Winter | 841 | 866 | 4,129 | 5,836 | 29.5 | 53.0 |
|  | 2013 | Spring | 2,544 | 1,748 | 4,948 | 9,240 | 22.9 | 38.6 |
|  | 2013 | Summer | 259 | 147 | 743 | 1,149 | 33.4 | 51.0 |
| *Super-population* | 2010*–*13 | All | 7,075 | 5,381 | 14,446 | 26,902 | 26.4 | 46.4 |
