# Supplementary material for: Challenges of implementing Mark-recapture studies on poorly marked gregarious delphinids
Source: PLoS One. 2018 Jul 11;13(7):e0198167. doi: 10.1371/journal.pone.0198167 (PMC6040702; doi:10.1371/journal.pone.0198167)
Supplement: S3 Table — Results are also included from the global test (GT; test 2+3). GOF tests were conducted for highly distinctive individuals (D1) only, and highly distinctive and distinctive individuals (D1 & D2) combined. Test 3.SR was re-run excluding the first capture of each individual and results are shown in italics. Values in bold indicate significance. Abbreviations: nick distinctiveness (ND), variance inflation factor (c^) and not applicable (na). (DOCX) [file pone.0198167.s008.docx]

**S3 Table**

| **ND** | **GOF values** | **2.CL** | **2.CT** | **3.SM** | **3.SR** | ***3.SR***  ***Re-run*** | **GT** | $\hat{\boldsymbol{c}}$ |
| --- | --- | --- | --- | --- | --- | --- | --- | --- |
| ***D1*** | Statistic | na | -1.334 | na | 5.335 | *1.419* | na | 1.14 |
|  | *P*-value | na | 0.182 | na | <0.0001 | *0.156* | na |  |
|  | χ^2^ | 25.220 | 24.405 | 20.873 | 46.181 | *5.644* | 116.679 |  |
|  | df | 24 | 11 | 19 | 12 | *8* | 66 |  |
|  | *P*-value | 0.394 | **0.011** | 0.344 | **<0.0001** | *0.687* | **<0.001** |  |
| ***D1 & D2*** | Statistic | na | -2.349 | na | 8.833 | *1.587* | na | 1.28 |
|  | *P*-value | na | 0.019 | na | <0.001 | *0.056* | na |  |
|  | χ^2^ | 46.565 | 38.817 | 27.255 | 118.919 | *14.947* | 231.557 |  |
|  | df | 32 | 11 | 23 | 12 | *8* | 78 |  |
|  | *P*-value | **0.046** | **<0.0001** | 0.245 | **<0.001** | *0.060* | **<0.001** |  |
